# RIPK1 can mediate apoptosis in addition to necroptosis during embryonic development

**DOI:** 10.1038/s41419-019-1490-8

**Published:** 2019-03-13

**Authors:** Xuhua Zhang, John P. Dowling, Jianke Zhang

**Affiliations:** Department of Microbiology and Immunology, Sidney Kimmel Cancer Center Thomas Jefferson University, 233S. 10th St, Philadelphia, PA 19107 USA

## Abstract

RIPK1 has emerged as a key effector in programmed necrosis or necroptosis. This function of RIPK1 is mediated by its protein serine/threonine kinase activity and through the downstream kinase RIPK3. Deletion of RIPK1 prevents embryonic lethality in mice lacking FADD, a signaling adaptor protein required for activation of Caspase 8 in extrinsic apoptotic pathways. This indicates that FADD-mediated apoptosis inhibits RIPK1-dependent necroptosis to ensure successful embryogenesis. However, the molecular mechanism for this critical regulation remains unclear. In the current study, a novel mouse model has been generated, by disrupting a potential caspase cleavage site at aspartic residue (D)324 in RIPK1. Interestingly, replacing D324 with alanine (A) in RIPK1 results in midgestation lethality, similar to the embryonic defect in FADD^−/−^ mice but in stark contrast to the normal embryogenesis of RIPK1^−/−^ null mutant mice. Surprisingly, disrupting the downstream RIPK3 alone is insufficient to rescue RIPK1^D324A/D324A^ mice from embryonic lethality, unless FADD is deleted simultaneously. Further analyses reveal a paradoxical role for RIPK1 in promoting caspase activation and apoptosis in embryos, a novel mechanism previously unappreciated.

## Introduction

Apoptosis is a major form of programmed cell death (PCD) and is executed by Caspases^1^. When PCD signaling pathways become dysregulated, developmental defects can occur at embryonic or postnatal stages. While apoptosis has been studied since the 1970s, necroptosis is a recently described form of PCD that is usually kept latent^2–5^. When apoptosis is disrupted, cell death signaling skews towards necroptosis, in which receptor interacting protein kinase 1 (RIP, RIP1 or RIPK1) and RIPK3 serve as key signaling effectors^6–10^. These two protein serine/threonine kinases interact with one another via their RIP homotypic interaction motif. This results in phosphorylation of both RIPK1 and RIPK3, leading to recruitment and activation of the mixed lineage kinase domain like (MLKL) protein. Once activated, MLKL translocates to and disrupts the plasma membrane^11,12^. Loss of membrane integrity during necroptosis results in the release of cellular contents, leading to inflammatory responses^13^.

In the immune system, PCD is required for maintaining homeostasis and suppression of autoimmunity^14^. The extrinsic pathway is triggered by the death receptors (DRs) including Fas and TNFR1, in which the FADD adaptor recruits Caspase 8, leading to apoptosis. RIPK1 has long been studied as a mediator of NFκB activation during pro-survival and pro-inflammatory signaling, until it became evident that RIPK1 also plays a role in an alternative death pathway, necroptosis, especially when apoptosis is compromised in various cell lines^6,7,15^. Studies by us and others provide evidence that RIPK1 and RIPK3-mediated necroptosis indeed occurs in vivo^16–19^, which helps explain the initial paradoxical observations of embryonic lethality in FADD^−/−^ or Caspase 8^−/−^ mice^20–22^. Moreover, RIPK1-mediated necroptosis leads to defect in mature T and B lymphocytes lacking FADD or Caspase 8^16,23,24^. In total, these data indicate that successful embryogenesis and normal TCR-induced proliferative responses require FADD-mediated suppression of RIPK1 and RIPK3-dependent necroptosis in vivo.

RIPK1-mediated necroptosis also occurs in neuronal cells, which leads to neurodegenerative pathologies^3^. Apoptotic cells are engulfed by phagocytic cells, which prevents spillage of intracellular contents, and thus limits tissue damage and inflammation^14,25,26^. Therefore, there is a clear benefit for avoiding necrosis. Currently, it remains unclear how FADD-mediated signaling keeps RIPK1-mediated necroptosis latent. One possibility is that RIPK1 is disabled via cleavage by Caspase 8 activated through FADD. Indeed, an earlier study indicated aspartic acid residue (D)324 of RIPK1 being targeted by Caspase 8^27^. However, this study argues that cleavage of RIPK1 by Caspase 8 promotes apoptosis, and there is currently a lack of definitive in vivo evidence to support this model or suggest an alternative mechanism. To address this paradox, we have developed a novel mouse model using CRISPR/Cas9-mediated gene editing to inactivate a predicted caspase cleavage site within the intermediate domain of RIPK1. Our data reveals a new mechanism in the regulation of RIPK1, indicating that RIPK1 resistance to Caspases not only facilitates necroptosis, but also promotes apoptosis in mouse embryos.

## Results

### Targeting the predicted Caspase 8 cleavage site in RIPK1 through CRIPSR/Cas9-mediated gene editing

A potential mechanism for suppression of RIPK1-dependent necroptosis is that FADD-mediated activation of caspases may lead to cleavage of RIPK1. A previous in vitro study indicated that RIPK1 could be a target of Caspase 8^27^. In particular, Caspase 8 appears to cleave RIPK1 in vitro at aspartic acid (D) residue 324. To test this potential mechanism in vivo, we generated a novel mouse model in which D324 in RIPK1 was replaced with alanine (A) through the CRISPR/Cas9-mediated gene editing strategy^28^. DNA sequencing analysis of the founder mice in the C57BL/6 background confirmed the resulting D324A mutation (Supplementary Fig. S1). We found that the heterozygous RIPK1^+/D324A^ founders were fertile, and when backcrossed with C57BL/6 mice, transmitted the mutant allele to the offspring. We found that heterozygous RIPK1^+/D324A^ mutant mice contained normal numbers of T cells and B cells in the primary and secondary lymphoid organs (data not shown). This indicates that the D324A mutation does not impose a dominant-negative effect.

**Fig. 1:**
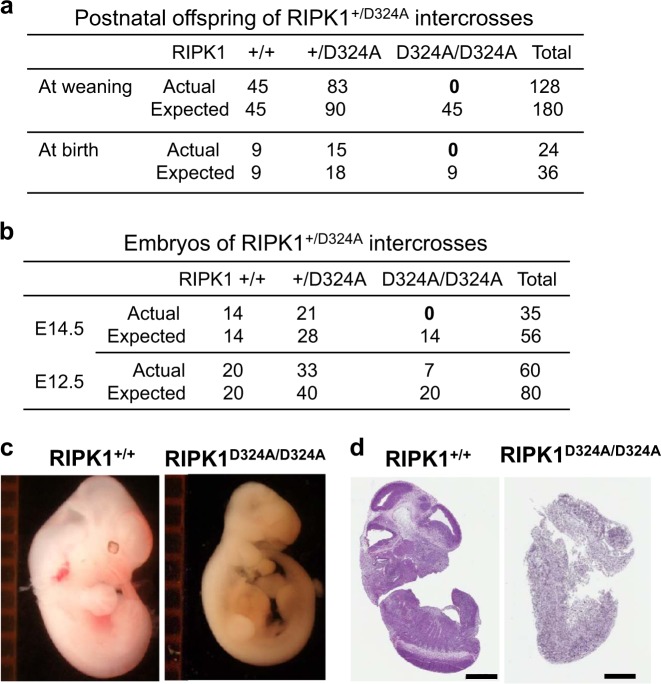
Analysis of the impact of the RIPK1 D324A mutation on postnatal and embryogenic development in mice. RIPK1^+/D324A^ heterozygous mutant mice undergo normal development, and were intercrossed. Genetic analyses of the offspring at weaning age or at birth (**a**), and at the indicated gestation stages (**b**), were performed. The RIPK1^D324A/D324A^ homozygous mutants were detected at E12.5, but not at E14.5, at birth or at weaning age. Representative images of whole embryos (**c**) and H&E staining of embryo sections (**d**) of mutant and wild type control embryos at E12.5. The ruler division in mm is shown to the left of embryos in (**c**). Scale bars in (**d**) = 1 mm

We then carried out intercrosses of the heterozygous RIPK1^+/D324A^ mice. Interestingly, no homozygous RIPK1^D324A/D324A^ mutant mice were detected in the offspring at the weaning age (Fig. 1a, top) or at birth (Fig. 1a, bottom). This data indicates that RIPK1^D324A/D324A^ mutant mice die before birth. Therefore, we performed timed mating analyses, but again found no homozygous RIPK1^D324A/D324A^ mutant embryos at embryonic (E) day 14.5 from intercrosses of heterozygous RIPK1^+/D324A^ mutant mice (Fig. 1b, top). Further analysis of earlier stages showed that RIPK1^D324A/D324A^ embryos were present at E12.5, albeit at lower than the predicted Mendelian ratios (Fig. 1b, bottom). When compared with wild type control E12.5 embryos, the RIPK1^D324A/D324A^ mutant E12.5 embryos had a clear developmental defect (Figs. 1c, d). These data indicate that the D324A mutation in RIPK1 results in embryonic lethality around E12.5. This observation is in sharp contrast to normal embryogenesis in RIPK1^−/−^ null mutant mice, as previously reported^16^.

### RIPK1^D324A/D324A^ embryonic death is partially dependent on RIPK3

**Fig. 2:**
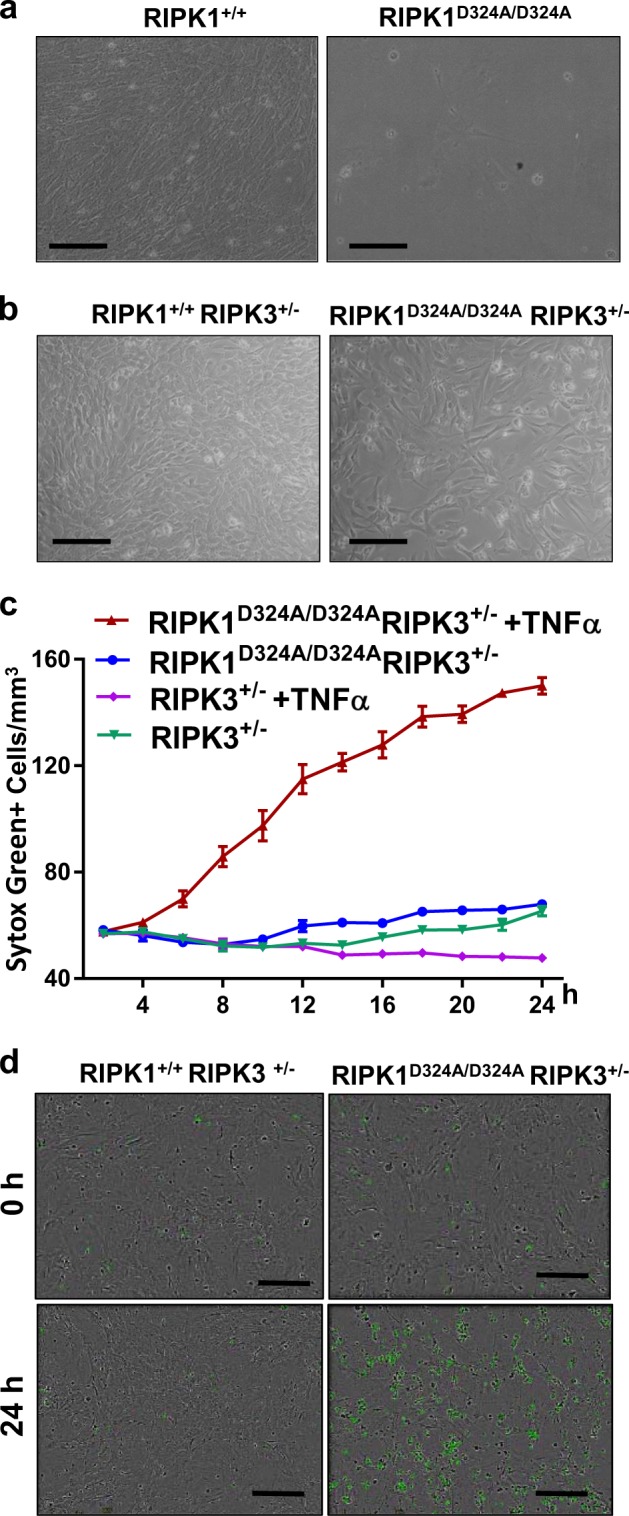
The RIPK1 D324A mutation leads to hypersensitivity to cell death responses. Images of MEFs with indicated genotypes prepared from RIPK1^+/D324A^ mouse intercross **a** or from RIPK1^+/D324A^ RIPK3^+/−^ mouse intercrosses (**b**). MEF cells of the indicated genotypes were cultured in the presence of SYTOX Green (50 nM), with or without TNFα (50 ng/ml) for 24 h (**c**). Real-time analysis was performed with an IncyCyte unit instrument. Dying cells are indicated as SYTOX Green^+^/mm^2^) and plotted against the indicated time-points. SEM were indicated by errors bars. **d** Real-time imaging analysis of MEFs treated with TNFα (50 ng/ml) and SYTOX Green (50 nM). Increased cell death is indicated by higher numbers of SYTOX Green^+^ cells in RIPK1^D324A/D324A^ RIPK3^+/−^ MEF, compared to RIPK1^+/D324A^ RIPK3^+/−^ control. The data represent at least three independent experiments. Scale bars in (**a**) and (**b**) = 100 μm, and in (**d**) = 300 μm

To facilitate further analysis, we prepared mouse embryonic fibroblasts (MEFs). Interestingly, D324A mutant MEFs displayed dramatically poor viability in culture (Fig. 2a) and we were unable to obtain a sufficient number of cells for further analysis. However, we found that deletion of one allele of RIPK3 improved viability of D324A mutant MEFs (Fig. 2b). We then performed real-time analysis of cell death responses using an IncuCyte system, and found that RIPK1^D324/D324^ RIPK3^+/−^ MEFs were highly sensitive to cell death induction by TNFα over a 24 h period, as shown by uptake of Sytox Green, whereas RIPK1^+/+^ RIP3K^+/−^ control MEFs were highly resistant to the cell death induction (Fig. 2c). Snapshot images of cell death at 0 h and 24 h post TNFα treatment also allowed for direct visualization of the greatly increased cell death in RIPK1^D324/D324^ RIPK3^+/−^ MEFs, compared with RIPK1^+/+^ RIPK3^+/−^ control MEFs (Fig. 2d).

**Fig. 3:**
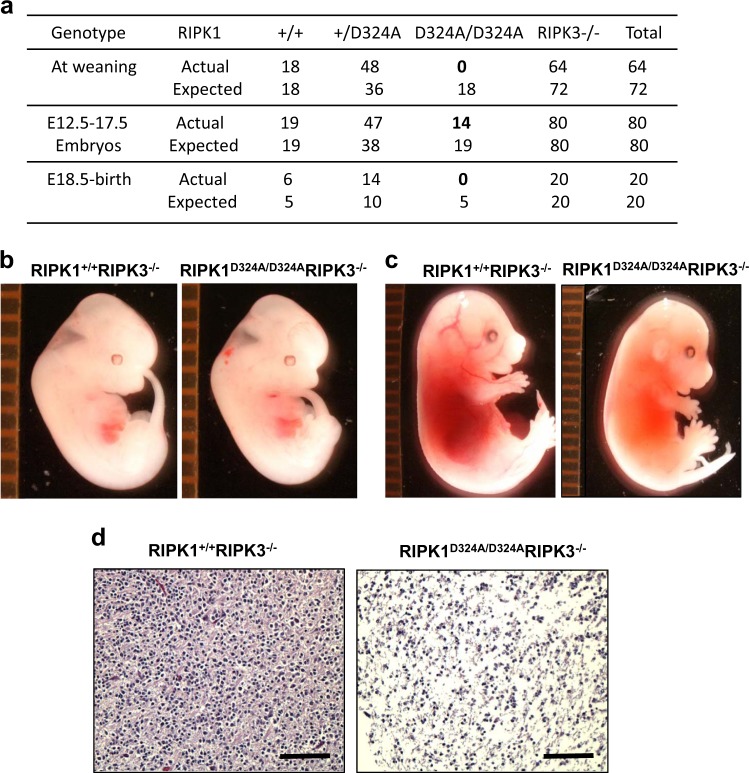
RIPK3-mediated necroptosis does not entirely explain the embryonic lethality in RIPK1^D324A/D324A^ mice. Genetic analysis indicated that RIPK3 deletion was unable to correct the developmental defect, because no viable RIPK1^D324A/D324A^ RIPK3^−/−^ mice were detected at weaning age (**a**, upper rows). Analyses of embryos from timed mating show that RIPK1^D324A/D324A^ RIPK3^−/−^ embryos can be found as late as E17.5 (**a**, lower rows). Images of embryos of indicated genotypes at E12.5 (**b**) and E17.5 (**c**). RIPK1^D324A/D324A^ RIPK3^−/−^ embryos appear normal at E12.5 (**b**), unlike the defect in E12.5 RIPK1^D324A/D324A^ (Fig. 1c). However, severe defect in RIPK1^D324A/D324A^ RIPK3^−/−^ embryos became apparent at E17.5 (**c**). Histological analysis via H&E staining of the embryos at E17.5 revealed major cell death in the RIPK1^D324A/D324A^ RIPK3^−/−^ embryo, contrasting the normal histology in RIPK1^+/+^ RIPK3^−/−^ control (**d**). Tissues shown represent the fetal liver areas. The ruler division in mm is shown to the left of embryos in (**b**), (**c**). Scale bars in (**d**) =100 μm.

To determine whether embryonic death in RIPK1^D324A/D324A^ mice is due to RIPK3-mediated necroptosis, RIP1K^+/D324A^ mice were crossed to mice lacking RIPK3. The resulting RIPK1^+/D324A^ RIPK3^−/−^ mice were intercrossed and genetic analysis of the offspring was performed. At weaning age, we detected no viable RIPK1^D324A/D324A^ RIPK3^−/−^ double mutant mice (Fig. 3a, top). Therefore, we analyzed embryos generated from RIPK1^+/D324A^ RIPK3^−/−^ mouse intercrosses and found that RIPK1^D324A/D324A^ RIPK3^−/−^ double mutant embryos were present from E12.5 to E17.5 (Fig. 3a, bottom). Although RIPK1^D324A/D324A^ RIPK3^−/−^ double mutant embryos appeared normal at E12.5 (Fig. 3b), they displayed an apparent defect by E17.5 (Fig. 3c), as compared to RIPK1^+/+^ RIPK3^+/+^ control embryos. Histological analysis revealed cell death at E17.5 in RIPK1^D324A/D324A^ RIPK3^−/−^ embryos, compared to RIPK1^+/+^ RIPK3^−/−^ control embryos (Fig. 3d). In total these data indicate that RIPK3-mediated necroptosis is partially responsible for death in RIPK1^D324A/D324A^ embryos.

### Analysis of RIPK3-independent pathway unleashed in RIPK1^D324A/D324A^ mice

**Fig. 4:**
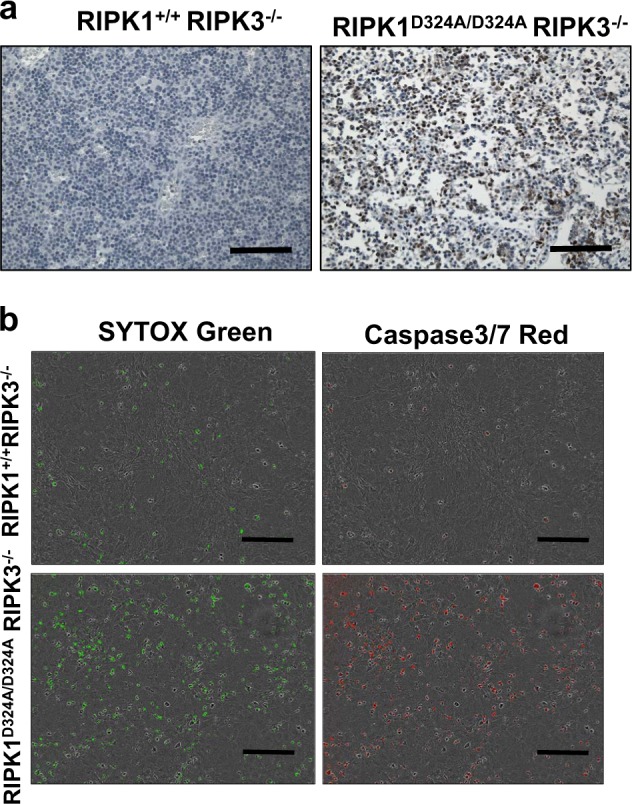
Caspase activities were greatly elevated in RIPK1^D324A/D324A^ RIPK3^−/−^ embryos. Immunohistochemistry analyses of E17.5 embryos of the indicated genotypes through staining with antibodies specific to cleaved Caspase 3 (**a**). Tissues shown represent the fetal liver areas. MEFs cells were treated with TNFα (50 ng/ml), and SYTOX Green (50 μM) was added to detect cell death (**b**). At 14 h, Caspase-3/7 red (5 μM) was added and two-color, real-time imaging was performed using an IncuCyte system at 24 h. Increased cell death (Green^+^, lower left) correlates with increased Caspase 3/7 activities (red^+^, lower right) in RIPK1^D324A/D324A^ RIPK3^−/−^ MEFs, when compared with RIPK1^+/+^ RIPK3^−/−^ control MEFs. Scale bars in (**a**) = 100 μm, and in (**b**) = 300 μm

It is interesting that lack of RIPK3 fails to restore normal embryogenesis in RIPK1^D324A/D324A^ mice, indicating that a RIPK3-independent pathway is also responsible in part for death at E17.5. During further analysis of the defect in RIPK1^D324A/D324A^ mice, we performed immunohistochemistry (IHC) assays and found that Caspase activities were greatly elevated in RIPK1^D324A/D324A^ RIPK3^−/−^ embryos, compared to RIPK1^+/+^ RIPK3^−/−^ embryo control (Fig. 4a). We also analyzed MEFs treated with TNFα using the IncuCyte system for real-time simultaneous detection of cell death and caspase activities. RIPK1^D324A/D324A^ RIPK3^−/−^ MEFs were hypersensitive to TNFα-induced death, as indicated by Sytox Green uptake (Fig. 4b, left). However, since RIPK3-mediated necroptosis is blocked in RIPK1^D324A/D324A^ RIPK3^−/−^ MEFs, an alternative form of cell death is occurring. Agreeing with IHC analysis (Fig. 4a), dramatically higher levels of caspase 3/7 activities were detected in RIPK1^D324A/D324A^ RIPK3^−/−^ MEFs than in control RIPK1^+/+^ RIPK3^−/−^ MEFs after TNFα treatment (Fig. 4b, right). These data in total suggest a skewing towards apoptotic responses in embryonic cells when RIPK1 is resistant to Caspase 8 via the D324A point mutation and necroptosis is blocked through knockout of RIPK3.

**Fig. 5:**
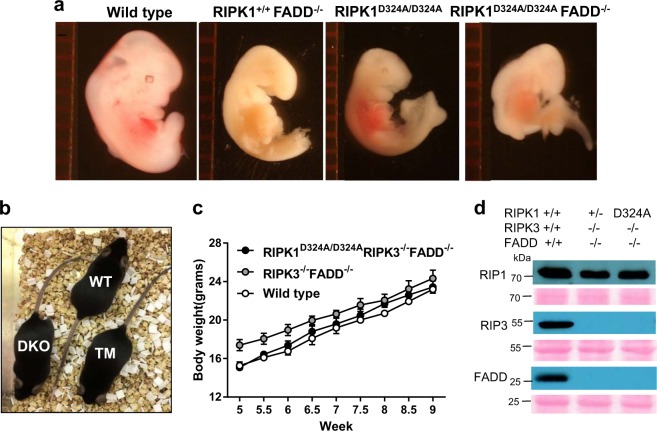
Normal development is restored by simultaneous deletion of both FADD and RIPK3 in RIPK1^D324A/D324A^ mice. Analysis was performed to determine the effect of disrupting FADD-mediated apoptosis. Defects in the three mutant E12.5 embryos of the indicated genotypes are similar, contrasting normal embryos in the wild type control (**a**). The ruler division in mm is shown to the left of embryos. At two-month age, RIPK1^D324A/D324A^ RIPK3^−/−^ FADD^−/−^ (TM) mice are indistinguishable in appearance (**b**) and in body weights (**c**) from the wild type (WT) and RIPK3^−/−^ FADD^−/−^ (DKO) control mice. Western blot analysis of total splenocytes confirming presence or absence of RIPK1, RIPK3, and FADD proteins (**d**). Ponceau *S* staining (pink) was performed as protein loading and transfer control

FADD recruits and facilitates Caspase 8 activation during DR-mediated signaling. Given elevated Caspase activation in RIPK1^D324/D324A^ embryos, it is possible that the D324A mutation in RIPK1 facilitates extrinsic apoptosis. To test this, the FADD knockout allele was crossed into RIPK1^D324A/D324A^ mice. However, deletion of FADD provides little improvement to the development of RIPK1^D324A/D324A^ embryos and vice versa. RIPK1^D324A/D324A^ FADD^−/−^, RIPK1^D324A/D324A^ FADD^+/+^ and FADD^−/−^ mutant embryos displayed similar developmental blockage (Fig. 5a).

We then determined the effect of simultaneous inactivation of both RIPK3 and FADD. To this end, RIPK1^+/D324A^ RIPK3^+/−^ FADD^+/−^ triple heterozygous (THZ) mutant mice were intercrossed. This mating produced no viable RIPK1^D324A/D324A^ RIPK3^−/−^ mice containing one or both alleles of endogenous FADD. However, viable RIPK1^D324A/D324A^ FADD^−/−^ RIPK3^−/−^ triple mutant mice were detected at weaning age (Fig. 5b). Western blot analysis of total splenocytes confirmed the presence or absence of RIPK1, RIPK3, and FADD protein (Fig. 5d). The resulting triple mutant mice reached adulthood with weight gain similar to wild type control mice as well as FADD^−/−^ RIPK3^−/−^ (DKO) mice (Fig. 5c), and they were fertile. In total, these data indicate that the D324A mutation of RIPK1 promotes both FADD-dependent apoptosis and RIPK3-dependent necroptosis, thus leading to embryonic lethality at midgestation stages, similar to FADD^−/−^ embryonic defect. This is in sharp contrast to RIPK1^−/−^ null mice, which undergo normal embryonic development but die soon after birth.

### The impact of RIPK1 D324A mutations on lymphoid compartment

**Fig. 6:**
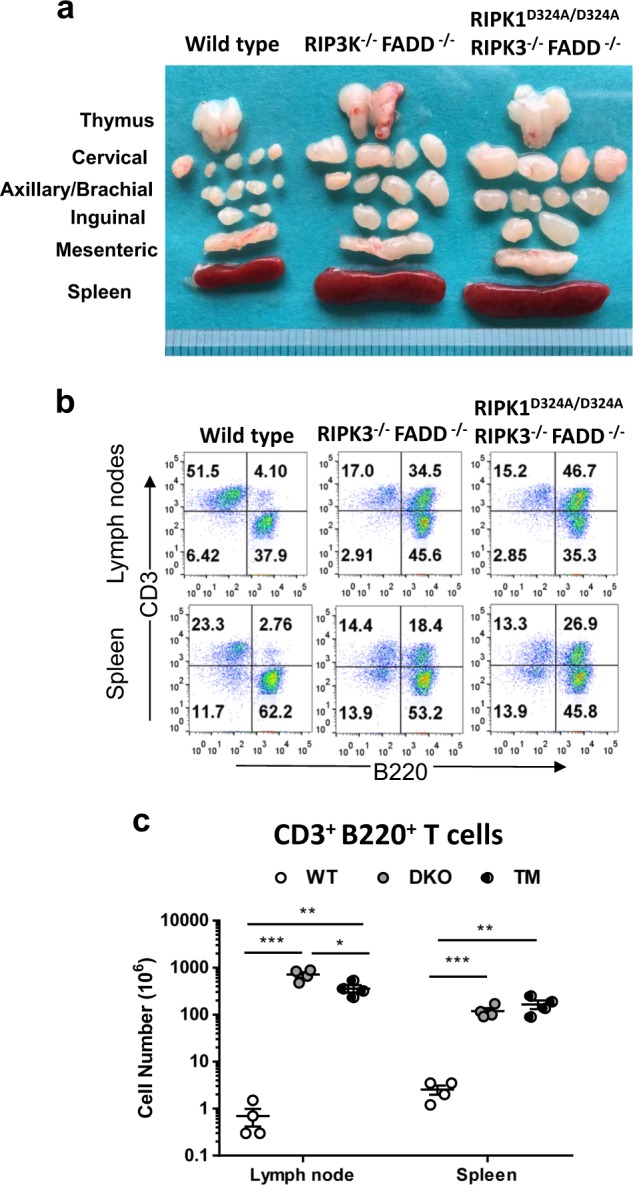
Analysis of the lymphoid compartments in TM mice. Representative images of the thymus, lymph nodes, and spleen of 3-month-old mice of the indicated genotypes show that the TM mice develop the signature *lpr* phenotype, comparable to that in DKO mice, as indicated by lymphadenopathy and splenomegaly (**a**). The ruler division in mm is shown to the bottom. Wild type control is shown (left). Two color flow cytometry analysis show that the typical CD3^+^ B220^+^ T cell population accumulates in the peripheral lymphoid organs in TM and DKO mice, contrasting the wild type control mouse (**b**). Scatter plots of total number of CD3^+^B220^+^ T cells of 3-month-old mice of the indicated genotypes (**c**). ^*^p < 0.05; ^**^p < 0.01; ^***^p < 0.001; *ns* not significant. Wild type (WT), double knockout mouse (DKO), and triple mutant (TM) mice are the same as in (**a**), (**b**)

Earlier studies have shown that disruption of the function of FADD by using a FADD-DN mutant or by deletion of FADD in aged RIPK3^−/−^ mice leads to a greatly exaggerated *lpr*-like symptom^16,19,24^. We found that the *lpr* phenotype, characterized as lymphadenopathy and splenomegaly, became apparent in FADD^−/−^ RIPK3^−/−^ double knockout (DKO) mice past 3 months of age (Fig. 6a, middle). A similar *lpr-*like phenotype was present in RIPK1^D324A/D324A^ FADD^−/−^ RIPK3^−/−^ triple mutant mice, compared to wild type control mice (Fig. 6a). This signature *lpr* phenotype was comparable between TM mice and DKO mice.

Flow cytometric analyses shows that DKO mice contain the unique subset of CD3^+^ T cells that express the B cell marker B220 (Fig. 6b), a signature phenotype of the Fas^−/−^ *lpr* mutant mice. Furthermore, these T cells lose the expression of CD4 and CD8 (Supplementary Fig. S3a). When absolute numbers of this peculiar T cell population were determined, DKO and TM mice at ages 3–6 months contain dramatically higher numbers of CD3^+^B220^+^ T cells (Fig. 6c). Moreover, the numbers of conventional T cells CD3^+^B220^−^ T cells and B cells are also significantly increased in the lymph nodes in DKO and TM mice (Supplementary Figs. S3b,c). In young adult mice (6 week old), the thymus, spleen and lymph nodes of RIPK1^D324A/D324A^ FADD^−/−^ RIPK3^−/−^ TM mice were similar in size and weight to that of FADD^−/−^ RIPK3^−/−^ DKO or wild type control mice (Supplementary Fig. S4a). Flow cytometric analysis revealed no obvious defect in the T cell and B cell lineages of TM mice, compared to DKO or wild control mice (Supplementary Figs. S4b, c).

### Death receptor signaling in RIPK1^D324A/D324A^ mutant mice

**Fig. 7:**
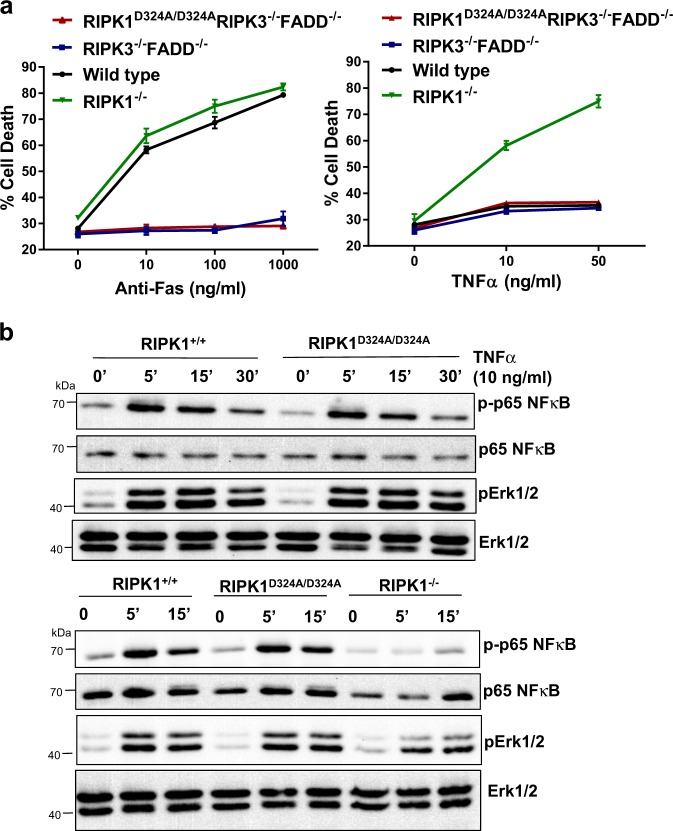
Analysis of the effect of the D324A mutation on DR-induced signaling pathways. **a** Thymocytes from mice of indicated genotypes were treated with various doses of anti-Fas antibodies (left) or TNFα (right) for 16 h and cell death was determined by PI uptake measured using a flow cytometer. **b** MEFs of the indicated genotypes were stimulated with TNFα (10 ng/ml) and induction of phosphorylation of p65 NFκB and Erk1/2 was analyzed by western blotting with the corresponding antibodies. Probing of the same membrane with antibodies for total NFκB or Erk1/2 protein was performed as protein loading/transfer control

The extrinsic cell death pathways mediated by DRs are essential for immune homeostasis, as indicated by the Fas^−/−^ *lpr* mouse model. We showed previously that when RIPK1 was absent, thymocytes underwent normal Fas-induced death, but became hypersensitive to TNFα-induced death^29^. In contrast, RIPK1^−/−^ FADD^−/−^ thymocytes were resistant to TNFα, similar to wild type thymocytes^16^. In order to determine the status of the DR pathways in TM mice, thymocytes were isolated and treated with various stimuli to initiate extrinsic cell death. Similar to DKO thymocytes, TM thymocytes were highly resistant to cell death responses induced by Fas (Fig. 7a). Unlike hypersensitivity in RIPK1^−/−^ thymocytes^16^, TM and DKO thymocytes were resistant to TNFα-induced cell death (Fig. 7b). TNFα can also trigger activation of the NFκB pathway and MAPK signaling events in a RIPK1-dependent manner. However, we found that these pathways were not affected in RIPK1^D324A/D324A^ MEFs cells treated with TNFα (Fig. 7b), indicating that the D324A mutation leads to a selective loss of function for RIPK1, essential for mouse embryogenesis. In contrast, loss of the entire RIPK1 protein results in severely impaired activation of NFκB and MAPKs Erk1/2 activation (Fig. 7b, bottom).

## Discussion

During embryonic development, RIPK1 can promote necroptosis, which is suppressed by FADD. This regulatory mechanism was demonstrated by our previous data showing that ablation of RIPK1 rescues FADD^−/−^ mice from embryonic lethality^16^. The resulting FADD^−/−^ RIPK1^−/−^ DKO mice undergo normal embryonic development. The current study aims to understand the mechanism for how RIPK1-mediated necroptosis in embryos is suppressed by FADD, an adaptor for Caspase 8 activation. To this end, we performed gene editing of RIPK1 in mice in an in vivo analysis of a potential mechanism that RIPK1 could be cleaved by Caspase 8 activated through FADD. Our current study reveals novel insight by providing in vivo evidence that besides playing an effector role in necroptosis in embryos, RIPK1 may actually promote apoptosis.

Firstly, mutating the Caspase cleavage site D324 of RIPK1 led to embryonic lethality (Fig. 1). The fact that RIPK1^D324A/D324A^ mutant mice die much earlier than RIPK1^−/−^ null mice, but similar to FADD^−/−^ embryonic death at E12.5 suggests that the D324A mutation rendered RIPK1constitutively active and readily promoted RIPK3-dependent necroptosis. Indeed, severe loss of cells was seen in RIPK1^D324A/D324A^ embryos (Figs. 1b, c), and there was poor ex vivo survival of RIPK1^D324A/D324A^ MEFs (Fig. 2a). Remarkably, deleting one allele of RIPK3 greatly improves RIPK1^D324A/D324A^ MEF growth (Fig. 2b). Deleting both alleles of RIPK3 prolongs embryonic development to E17.5, indicating that RIPK1^D324A/D324A^ embryos die, due in part to RIPK3-mediated necroptosis. However, these embryos do not survive to birth (Fig. 3). This observation was unexpected, especially given that RIPK1^−/−^ or FADD^−/−^ RIPK1^−/−^ embryos undergo full term gestation, and no death occurs until after birth^16^.

A plausible explanation for this paradoxical observation is that the D324A mutation in RIPK1 skews response to another cell death pathway. To this end, we found elevated CC3 levels present in RIPK1^D324A/D324A^ RIPK3^−/−^ embryos and TNFα-treated MEFs (Fig. 4), indicating that apoptosis may also occur in RIPK1^D324A/D324A^ RIPK3^−/−^ embryos. In further support of this notion, we found that RIPK1^D324A/D324A^ RIPK3^−/−^ MEFs were hypersensitized to apoptotic death in responses to treatment with TNFα (Fig. 4b). However, deletion of FADD alone does not provide any improvement on embryogenesis (Fig. 5a). It is only when both FADD and RIPK3 are absent that normal development is ensured in RIPK1^D324A/D324A^ mice (Fig. 5b). This striking observation illustrated the unique power of the new RIPK1^D324A/D324A^ mouse model, which reveals a novel function of RIPK1 in protecting embryos from apoptosis. This newly discovered function of RIPK1 during embryogenesis has not been appreciated using the previously described RIPK1^−/−^, or FADD^−/−^ RIPK1^−/−^ or FADD^−/−^ RIPK1^−/−^ RIPK3^−/−^ mice. In total, we determined that Caspase cleavage of RIPK1 not only protects against necroptosis, but also RIPK1-dependent apoptosis.

In contrast to the perinatal lethality of FADD^−/−^ RIPK1^−/−^ mice^16^, FADD^−/−^ RIPK3^−/−^ DKO mice have normal embryonic and postnatal development by blocking not only RIPK3-dependent necroptosis, but also FADD-independent apoptosis^19^. RIPK1^D324A/D324A^ mice also survive to adulthood when both FADD and RIPK3 are knocked out (Fig. 5). These findings provide an additional platform to determine if caspase cleavage of RIPK1 has a cell death-independent role. To this end, the RIPK1^D324A/D324A^ FADD^−/−^ RIPK3^−/−^ TM and FADD^−/−^ RIPK3^−/−^ DKO mice are comparable to wild type mice at young adult age (Supplementary Fig. S4) and develop progressive lymphadenopathy and splenomegaly, when aged, displaying the *lpr*-like phenotype (Fig. 6 and Supplementary Fig. S3).

Although RIPK1 is isolated as a Fas-interacting protein, its absence does not impact Fas-induced apoptosis^30,31^. Instead, RIPK1 deficiency is most often associated with defective NFκB activation induced by TNFR1^32^. Deletion of TNFR1 does not apparently affect mouse development, but RIPK1 deficiency leads to perinatal lethality^31^. This paradox remained unresolved until recent studies showed that RIPK1^−/−^ mice can survive into late adulthood only when apoptosis is blocked by deletion of FADD or Caspase 8 and necroptosis is blocked by deletion of RIPK3^19,33–35^. This finding implies that RIPK1 suppresses both FADD/Caspase 8-mediated apoptosis and RIPK3-mediated necroptosis at the perinatal stage.

RIPK1 has several putative functional domains, and each has been proposed to play important roles in distinct signaling pathways. Our current study employs a targeted and specific approach to answer a question, untestable through the previous models including RIPK1^−/−^ mice lacking the entire RIPK1 protein. We found that although RIPK1^D324A/D324A^ cells are hypersensitive to both necroptosis and apoptosis induction by TNFα, the Fas-induced cell death pathway was not affected by the D324A mutation in RIPK1 (Fig. 7a). Furthermore, non-cell death pathways induced by TNFα, such as activation of the NFκB and MAPK signaling were also not impacted (Fig. 7b). In particular, our data revealed a novel role for RIPK1 in promoting apoptosis in embryos. Previous in vitro/ex vivo study suggested that RIPK1 cleavage by Caspase 8 facilitate apoptosis in cell lines^27^. Our current study provides the first direct in vivo evidence that FADD-Caspase 8 disable RIPK1 through cleavage at D324, and as a result, protect embryonic cells against not only necroptosis but also apoptosis. RIPK1-dependent apoptosis induced by TNFα has been previously shown to occur in other settings^32,36–42^. A more recent study shows that a highly ubiquitinated, insoluble form of RIPK1 is important for apoptotic signaling and identifies new players regulating RIPK1-dependent apoptosis^43^. Previous studies have shown that the inhibitor cFLIP not only blocks apoptosis, but also necroptosis^44^. One possibility is that a cFLIP-caspase 8 heterodimmer blocks not only RIPK1-dependent necroptosis^17^ but also RIPK1-dependent apoptosis via a caspase 8 homodimer. Interestingly, in proximity to D324, S321 can be phosphorylated by MK2, which helps suppress RIPK1-dependent apoptosis and necroptosis, as shown in recent studies^45,46^. Therefore, several regulatory mechanisms target the intermediate region of RIPK1. The implications of the current study are wide ranging and could be studied in the context of cancer by inducing necroptosis in cancer cells through preventing RIPK1 cleavage or autoimmune and inflammatory diseases, where RIPK1 cleavage could be induced to prevent necroptosis.

## Materials and methods

### Mice

A previously described strategy^28^ was employed to generate RIPK1^+/D324A^ founder mice, through CRISPR/Cas9-mediated gene editing in the C57BL/6 background using the guide RNA 5′-CCTGAATTTGACCTGCTCGGAGG-3′. RIPK3^−/−^ mice were kindly provided by Drs. Kim Newton and Vishva Dixit at Genentech^47^. FADD^+/−^ mice have been described in our previous studies^16,20^. All animal studies were approved by Institutional Animal Care and Use Committee (IACUC) at Thomas Jefferson University. Genotyping of RIPK1^+/D324A^ mice was performed using the primers: 5′-AGAATGTTTTCACTGCAGCATGAC-3′ and 5′-GGTACACAGACTCAGACACACATAC-3′ for the endogenous allele; 5′-AATGTTTTCACTGCAGCATGCT-3′ and 5′-GGTACACAGACTCAGACACACATAC-3′ for the D324A mutant allele. RIPK1^+/D324A^ mice were intercrossed and the resulting offspring at various embryonic stages and the weaning age were genotyped by PCR. For embryo analysis, timed mating were set up with RIPK1^+/D324A^ mice in the evening and females were checked in the morning. When vaginal plug was detected, embryos were designated E0.5. Embryos were isolated from pregnant mice at various gestation stages and genotypes were determined by PCR analyses. Genotyping information for additional gene alleles is shown in Supplementary Fig. S2.

### Cell culture

Mouse embryonic fibroblasts (MEFs) were isolated from E10.5 to E12.5 embryos. Embryos of the desired genotypes were placed in 24-well plates with trypsin (0.25%), chopped up with scissors or a scalpel blade. The resulting tissues pieces (1–2 mm^3^) were further sheared by pipetting up and down several times, and then incubated at 37 °C, 5% CO_2_ for 10 min. The digested embryonic tissues/cells were cultured in DMEM medium containing FBS (10%), L-Glutamine (2 mM), penicillin (100 U/mL), streptomycin (100 μg/mL), and β-mercaptoethanol (100 μM), at 37 °C with 5% CO_2_. Thymocytes and peripheral T cells were isolated from the spleen and lymph nodes and were cultured in RPMI medium containing FBS (10%), L-Glutamine (2 mM), penicillin (100 U/mL), streptomycin (100 μg/mL), and β-mercaptoethanol (5 μM).

### Immunohistochemistry analysis

Embryos were fixed in 10% buffered formalin for 2 days, were then washed 2–3 times with PBS and transferred to 70% ethanol, and finally embedded in paraffin. Sections were stained with hematoxylin and eosin or with antibodies specific for activated/cleaved caspase 3 (Cell Signaling Technology #9661).

### Flow cytometry

Lymph nodes, spleen and thymus were isolated during mouse dissection. Single cell suspension was prepared and red blood cells were depleted by hypotonic lysis. Cells were stained on ice for 30 min in PBS containing 3% BSA, 1 mM EDTA, and 0.05% sodium azide with fluorochrome-conjugated antibodies anti-CD3 (BD Biosciences, clone 17A2), anti-CD4 (BD Biosciences, clone GK1.5), anti-CD8 (Caltag, clone CT-CD8a), anti-B220 (Caltag, clone RA3–6B2). Samples were washed twice with PBS. Data was acquired on LSR II flow cytometric analyzer (BD Biosciences) and analyzed using FlowJo software (Treestar). Total cell numbers were counted on Countess Automated Cell Counter (Invitrogen) and lymphocyte cellularity was determined by multiplying the total cell number of indicated organ by percentage of CD3^+^ or B220^+^ cells within the organ obtained by flow cytometry.

### Cell death assays

Thymocytes (10^5^/well) were seeded in triplicate in 96-well flat-bottom plates in complete RPMI with various concentrations of anti-Fas antibody (BD Biosciences, clone Jo2) or TNFα. After incubation for 16 h, 1 μg/ml propidium iodide (PI) and the percentage of cell death was analyzed by flow cytometry as determined by PI^+^ cells.

### Western blotting analysis

The presence or absence of FADD, RIPK3, and RIPK1 protein was confirmed by western blotting. Total splenocytes were isolated from mice of the indicated genotypes. MEFs were stimulated with 10 ng/ml TNFα for indicated time points. Cell lysates were prepared in cold RIPA lysis buffer containing 50 mM Tris pH 8.0, 150 mM NaCl, 1% Nonidet P-40, 0.5% deoxycholate, 0.1% SDS, 1 mM phenylmethyl sulphonyl fluoride, and 1x Halt protease inhibitor cocktail (Thermo Scientific, #78430). Additional inhibitors were added when probing for phospho-specific proteins (50 mM β-glycerophosphate, 1 mM sodium vanadate, 10 mM sodium fluoride). Proteins (40 μg) were separated on a 10% SDS-PAGE gel and transferred to a nitrocellulose membrane. Proteins on the membrane were stained with Ponceau *S* (Sigma-Aldrich, #78376) as a loading/transfer control. Antibodies specific for FADD (generated in house), RIPK3 (ProSci, #2283), RIPK1 (BD Biosciences, #610459), p65 (Cell Signaling, #3034), p44/42 MAPK (Erk1/2) (Cell signaling, #9102) were incubated with the membrane overnight at 4 °C followed by streptavidin-horseradish peroxidase (HRP)-conjugated anti-rabbit (1/10,000,Vector laboratories #PI-1000) or anti-mouse antibodies (1/10,000, Vector laboratories #PI-2000). For analyses of TNF-induced signaling, membranes were blocked with 5% BSA in TBST for 1 h at room temperature and blotted overnight in 5% BSA in TBST containing antibodies specific for phosphorylated forms of p65 NFκB (Cell signaling, #3033S), Erk1/2(p42/44) (Cell signaling, #9106S). Membranes were incubated with HRP-conjugated secondary antibodies at room temperature for 1 h, followed by incubation with Western Lightning Plus-ECL reagent (Perkin Elmer, NEL105001EA). Chemiluminescent signals were detected using X-ray film or with ProteinSimple FluorChem M imaging system (ProteinSimple).

### Cell viability and caspase 3/7 activity

MEFs cells were seeded to 24-well plate (10^5^ cells/well). After overnight incubation (about 60% confluence), TNFα (50 ng/ml) and SYTOX^TM^ Green (50 nM, Invitrogen, S34860) and/or Caspase-3/7 red (5 μM, Essen bioscience, #4440) were added and real-time analyses of cell death responses were analyzed an IncuCyte instrument.

### Statistical analysis

Data is represented as mean ± Standard error of the mean (SEM). Student’s *t*-tests were performed using Prism software v7.03 (Graphpad Software, Inc.) to determine *p* values. A *p* value < 0.05 was considered significant. Sequence alignment was performed using Geneious Software (Geneious).

## Supplementary information

Supplemental Figure

Supplemental Figure legends
