# Supplementary material for: RIPK1 can mediate apoptosis in addition to necroptosis during embryonic development
Source: Cell Death Dis. 2019 Mar 13;10(3):245. doi: 10.1038/s41419-019-1490-8 (PMC6416317; doi:10.1038/s41419-019-1490-8)
Supplement: Supplementary file 1 — Supplemental Figure [file 41419_2019_1490_MOESM1_ESM.pdf]

Fig. S1

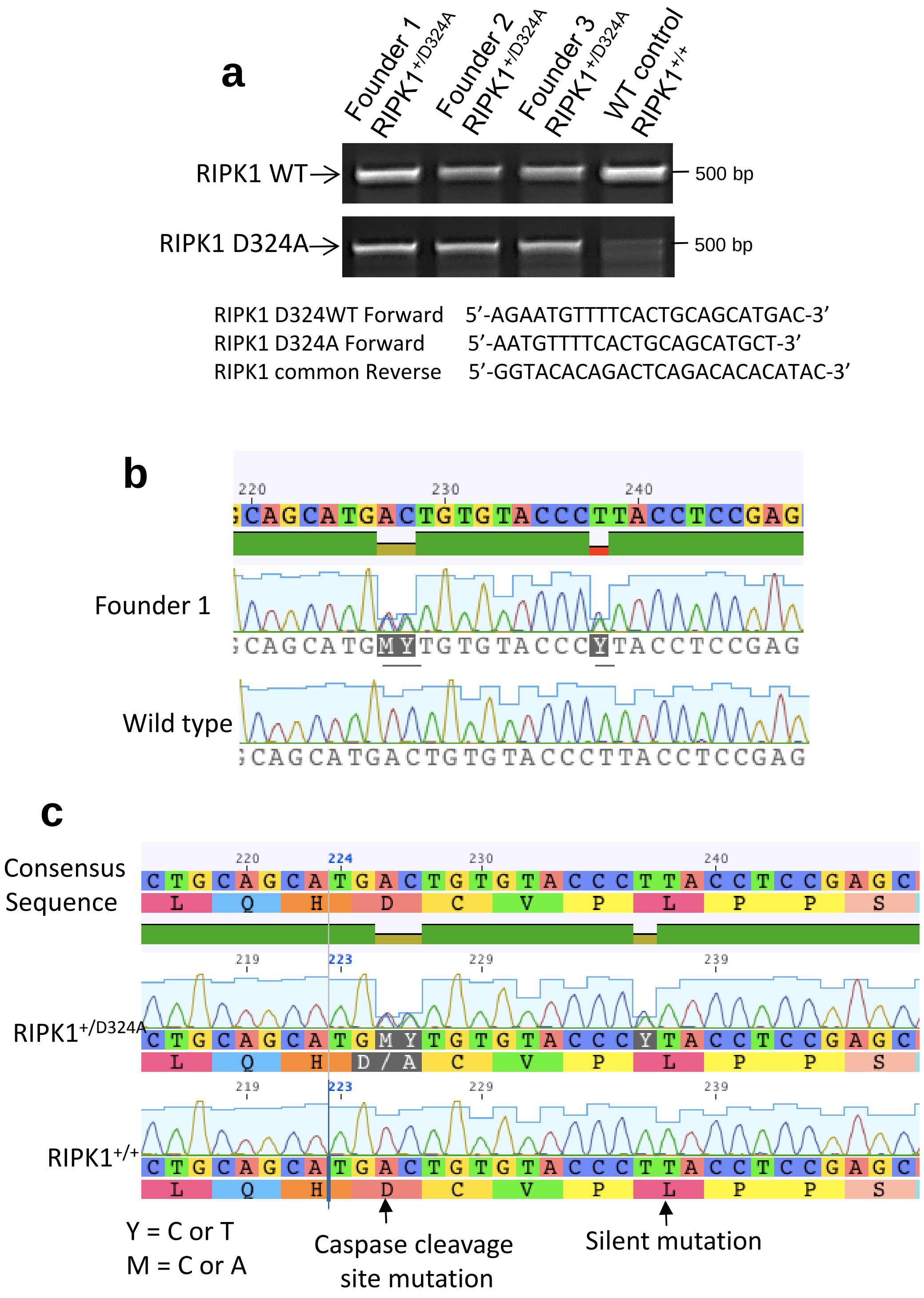
a
Founder 1 RIPK1+/D324A
Founder 2 RIPK1+/D324A
Founder 3 RIPK1+/D324A
WT control RIPK1+/+
RIPK1 WT
RIPK1 D324A
500 bp
500 bp
RIPK1 D324WT Forward 5'-AGAATGTTTTCACTGCAGCATGAC-3'
RIPK1 D324A Forward 5'-AATGTTTTCACTGCAGCATGCT-3'
RIPK1 common Reverse 5'-GGTACACAGACTCAGACACACATAC-3'
b
220
230
240
Founder 1
Wild type
c
Consensus Sequence
RIPK1+/D324A
RIPK1+/+
Y = C or T
M = C or A
Caspase cleavage site mutation
Silent mutation

Fig. S2

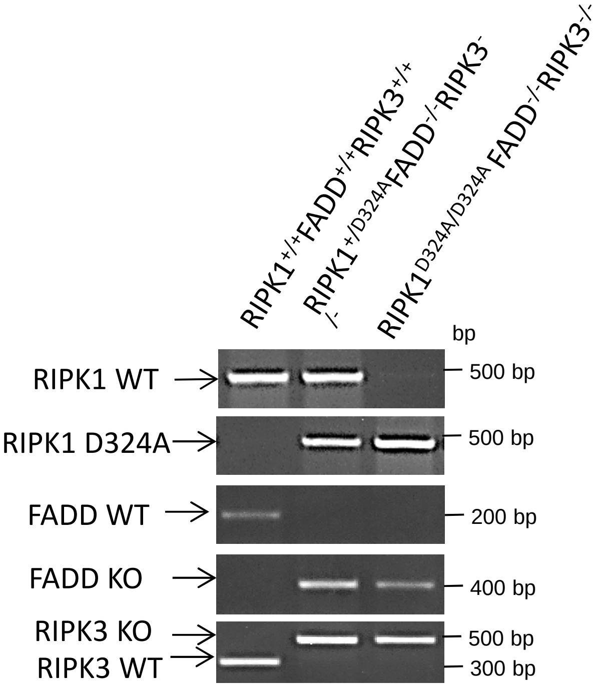

RIPK1 D324WT F 5’-AGAATGTTTTCACTGCAGCATGAC-3’
RIPK1 D324A F 5’-AATGTTTTCACTGCAGCATGCT-3’
RIPK1 common R 5’-GGTACACAGACTCAGACACACATAC-3’
FADD WT F 5’-ATG GAC CCA TTC CTG GTG CTG CTG -3’
FADD WT R 5’-CAG TAG ATC GTG TCG GCG CAG CG -3’
FADD KO F 5’-ACTGTAGTGCCCAGCAGAGACCAGC -3’
FADD KO R 5’-CGCTCGGTGTTCGAGGCCACACGC -3’
RIPK3 WT F 5’-CCAGAGGCCACTTGTGTAGCG-3’
RIPK3 KO F 5’-GCCTGCCCATCAGCAACTC-3’
RIPK3 common R 5’-CGCTTTAGAAGCCTTCAGGTTGAC-3’

Fig. S3

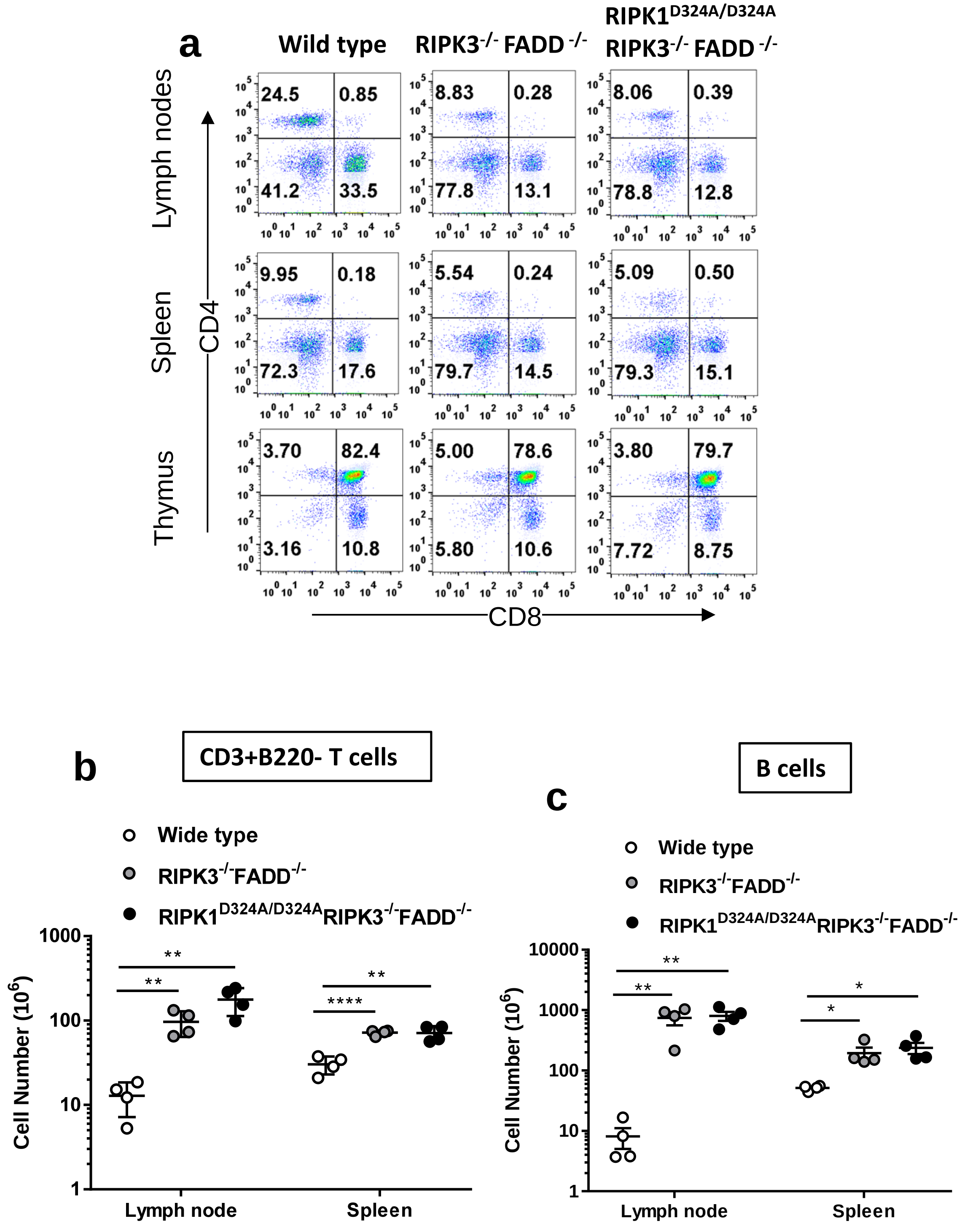
a
Wild type
RIPK3-/- FADD -/-
RIPK1D324A/D324A RIPK3-/- FADD -/-
Lymph nodes
Spleen
Thymus
CD4
CD8
24.5
0.85
41.2
33.5
8.83
0.28
77.8
13.1
8.06
0.39
78.8
12.8
9.95
0.18
72.3
17.6
5.54
0.24
79.7
14.5
5.09
0.50
79.3
15.1
3.70
82.4
3.16
10.8
5.00
78.6
5.80
10.6
3.80
79.7
7.72
8.75
b
CD3+B220- T cells
Wide type
RIPK3-/-FADD-/-
RIPK1D324A/D324ARIPK3-/-FADD-/-
Cell Number (10^6)
1000
100
10
1
Lymph node
Spleen
c
B cells
Wide type
RIPK3-/-FADD-/-
RIPK1D324A/D324ARIPK3-/-FADD-/-
Cell Number (10^6)
10000
1000
100
10
1
Lymph node
Spleen

Fig. S4

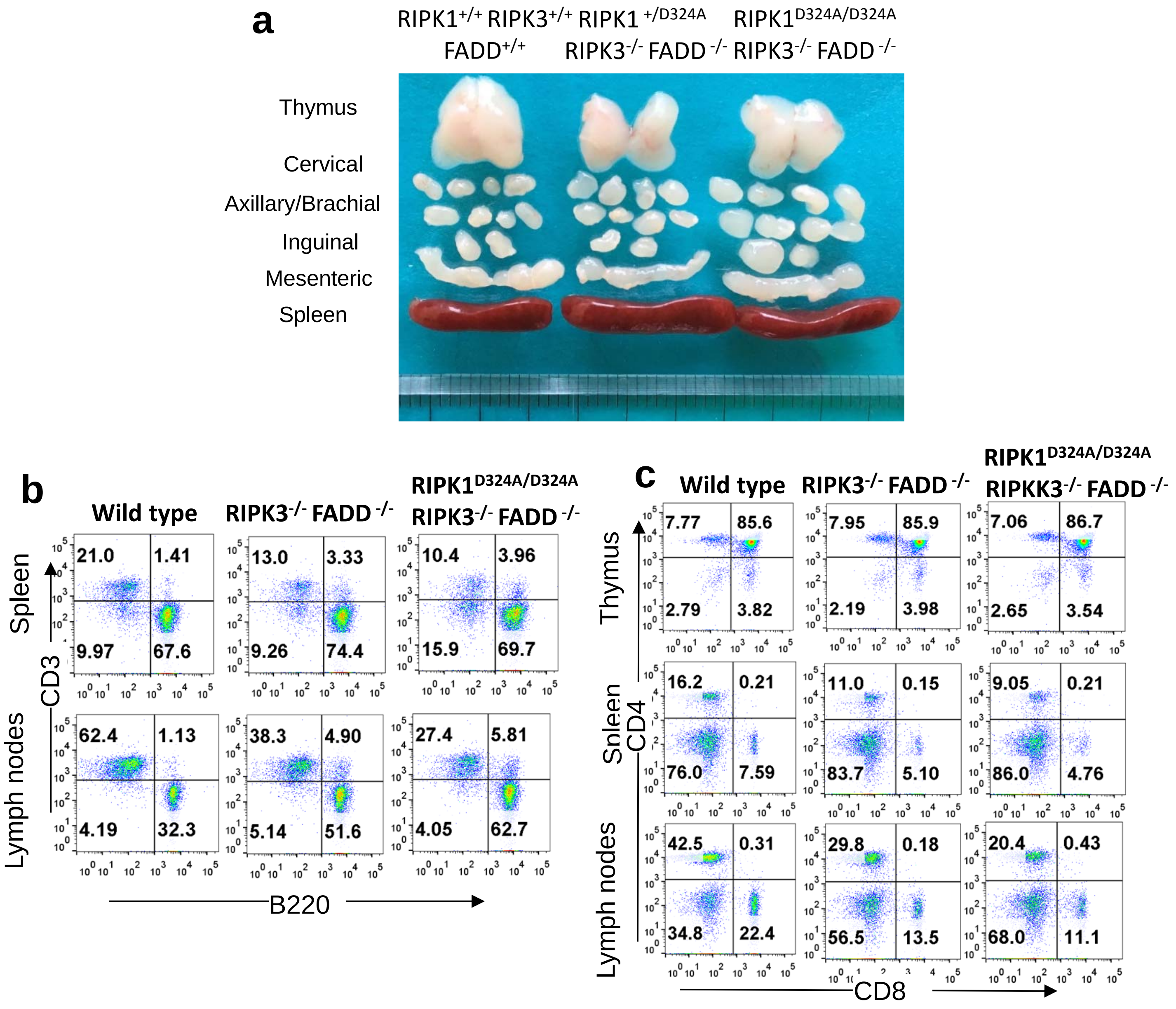
a
RIPK1+/+ RIPK3+/+ FADD+/+
RIPK1 +/D324A RIPK3-/- FADD -/-
RIPK1D324A/D324A RIPK3-/- FADD -/-
Thymus
Cervical
Axillary/Brachial
Inguinal
Mesenteric
Spleen
b
Wild type
RIPK3-/- FADD -/-
RIPK1D324A/D324A RIPK3-/- FADD -/-
Spleen
Lymph nodes
CD3
B220
21.0 1.41 9.97 67.6
13.0 3.33 9.26 74.4
10.4 3.96 15.9 69.7
62.4 1.13 4.19 32.3
38.3 4.90 5.14 51.6
27.4 5.81 4.05 62.7
c
Wild type
RIPK3-/- FADD -/-
RIPK1D324A/D324A RIPKK3-/- FADD -/-
Thymus
Spleen
Lymph nodes
CD4
CD8
7.77 85.6 2.79 3.82
7.95 85.9 2.19 3.98
7.06 86.7 2.65 3.54
16.2 0.21 76.0 7.59
11.0 0.15 83.7 5.10
9.05 0.21 86.0 4.76
42.5 0.31 34.8 22.4
29.8 0.18 56.5 13.5
20.4 0.43 68.0 11.1
