# Supplementary material for: RIPK1 can mediate apoptosis in addition to necroptosis during embryonic development
Source: Cell Death Dis. 2019 Mar 13;10(3):245. doi: 10.1038/s41419-019-1490-8 (PMC6416317; doi:10.1038/s41419-019-1490-8)
Supplement: Supplementary file 2 — Supplemental Figure legends [file 41419_2019_1490_MOESM2_ESM.docx]

**Supplementary Figure legend**

**Supplementary Fig. 1S. Genotype determination by PCR and DNA sequencing analysis of the founder mice to confirm the D324A mutation.** Genomic DNA extracted from mouse ear tissues was used as template in PCR amplification using the allele-specific primers listed (**a**). Genotypes was determined by sequencing of the DNA fragment amplified in (**b**). Predicted amino acid sequence of a founder and wild type mouse based on sequencing data alignment (**c**).

**Supplementary Fig. S2. Genotyping by PCR to detect various gene alleles of interests.** Ear tissue DNA was used as templates for amplification of the indicated gene alleles, using the primers listed.

**Supplementary Fig. S3. Analysis of lymphoid cells in RIPK1^D324A/D324A^ FADD^-/-^ RIPK3^-/-^** **mice.** Cells from the thymus, spleen and lymph nodes of 6-month-old mice of the indicated genotypes were stained with antibodies for CD4 and CD8 and analyzed by flow cytometry (**a**). Peripheral T cell numbers (**b**) and B cell numbers (**c**) of indicated genotypes at 6 month of age. At least 4 mice from each group were used to generate given data.^*^*p* < 0.05; ^**^*p* < 0.01; ^***^*p* < 0.001; ^****^*p* < 0.0001; ns, not significant.

**Supplementary Fig. S4. The lymphoid compartment in young adult mutant mice.** Due to the age-dependent *lpr*-like disease (Fig. 5 and Fig. S2), we analyzed younger TM mice. The thymus, spleen and lymph nodes of 6-week-old mice are similar in size among the indicated genotypes (**a**). The ruler division in mm is shown to the bottom. Flow cytometry analysis was performed and showed normal CD3^+^ vs. B220 **(b)** and CD4 vs. CD8 (**c**) population ratios.
